# Improvement of Photocatalytic Performance by Building Multiple Heterojunction Structures of Anatase–Rutile/BiOI Composite Fibers

**DOI:** 10.3390/nano12213906

**Published:** 2022-11-05

**Authors:** Dayu Li, Kai Xu, Chao Zhang

**Affiliations:** School of Mechanical Engineering, Yangzhou University, Yangzhou 225009, China

**Keywords:** photocatalysis, hydrothermal synthesis, multiple heterojunction structure, anatase–rutile/BiOI, composite fiber

## Abstract

In this study, multiple heterojunction structures of anatase–rutile/Bismuth oxyiodide (BiOI) composite fibers are designed by the combined method of electrospinning and hydrothermal techniques. The influence of different Ti/Bi atomic ratios ([Ti/Bi]) on the nanostructures and photocatalytic properties are investigated. It is found that the morphology of BiOI covered on the TiO_2_ fiber surface changed with [Ti/Bi] from nanosheets to submicron spheres structures. Additionally, the crystallization of the composite fibers including the phases of anatase, rutile, and BiOI is identified, theses phases are in close contact with each other, and the interfacial effects are helpful to form the multiple heterojunctions which lead to blue shifts on the chemical state of Ti. The absorption of visible light has been improved by compositing BiOI on TiO_2_, while the band gap values of the composite fibers are significantly reduced, which can enhance the generation and separation of electrons and holes. For the case of [Ti/Bi] = 1.57, the photodegradation rate of anatase–rutile/BiOI composite fibers is about 12 times that of pure TiO_2_. For the photocatalytic mechanism, the synergistic s-type heterojunctions increase the content of active oxides which have a positive effect on the degradation rate.

## 1. Introduction

Over the past decade, with the acceleration of industrialization, more and more serious water pollution problems have aroused people’s concern [1]. In our country, many sewage treatment methods such as photocatalytic degradation [2], electrocatalytic degradation [3,4], adsorption [5], thin film technology [6], and biodegradation [7], etc., are adopted to solve the problems. Among them, photocatalytic materials are widely used, because these special materials can directly apply solar energy, and have the advantages of no secondary pollution and convenient recycling. In recent years, it has become the main research focus. Except for removing pollutants, photocatalysis can also be used to solve the problem of energy shortage [8]. However, many photocatalysts can only absorb UV light (accounting for only 4–5% of solar energy). As an n-type semiconductor, TiO_2_ is a widely used photocatalytic material [9] with the characteristics of biological inertia, strong oxidation, non-toxicity, and no light corrosion. However, TiO_2_ has a relatively wide band gap (~3.1 eV) [10], and its photocatalytic degradation ability can only be activated under the excitation of ultraviolet light, so the utilization rate of sunlight is low.

With the deepening of semiconductor research, its related technology has been applied in more and more different fields, and semiconductor composite scheme has also been applied in TiO_2_ modification technology by many scholars. In order to solve the problem of poor photocatalytic activity of TiO_2_, the most commonly used methods are ion doping [11], surface modification [12], and semiconductor composite [13]. Semiconductor composite heterojunction formation is widely concerned as an effective method to improve photocatalytic activity. Compared with the single-phase photocatalyst, the formation of a polyheterojunction structure will increase the absorption of ultraviolet and visible light and increase the separation efficiency of photoelectrons and holes. This is because when one kind of semiconductor is combined with another kind of semiconductor, the small bandgap semiconductor absorbs a large amount of sunlight as a photosensitizer, and the photoelectrons on the stimulated small bandgap semiconductor surface can be transferred to another semiconductor, which effectively separates the photogenerated electrons from the holes and greatly improves the efficiency of photocatalysis [14]. In recent years, compounds such as bismuth halooxide BiOX (X = Cl, Br, I) have come into the view of researchers [15]. BiOX is generally used as ferroelectric materials and pigments, but studies have found that BiOX has good visible light and ultraviolet photocatalytic activity [16]. Compared with other bismuth halides, BiOCl has a relatively high band gap between 3.22 eV and 3.50 eV. Band gaps of BiOBr and BiOI are about 1.77 eV and 1.74 eV, respectively, which mainly absorb visible light [17]. Among new semiconductor photocatalysts, BiOI has a tetragonal crystal structure, is soluble in hydrochloric acid, insoluble in water, alcohol, etc., which has become a research hotspot because of its stable chemical properties, layered structure, non-toxic and low cost. However, BiOI is not perfect, its valence band is not positive enough, the separation efficiency of photogenerated electron holes is low, and micro-nanoparticles are prone to agglomeration, which is difficult to be recycled and reused [18,19,20,21]. So, due to the advantages and shortages of pure TiO_2_ and BiOX, some researchers have tried to mix these two materials to produce photocatalysts with high degradation efficiency. In the literature, it is found that the TiO_2_/BiOI p-n heterojunction can really improve the photocatalytic activity in the visible light wavelength range compared with the single BiOX and TiO_2_ phase [22,23,24]. Zhang et al. [25] synthesized TiO_2-X_/BiOI heterojunction by a simple hydrothermal synthesis method, its photocatalytic ability was characterized by the rate of H_2_ production to verify the utilization of light, which is higher than that produced by original material.

However, people also have to face the problems in practical applications as follows, (i) the powder recovery is difficult, and it is easy to cause secondary pollution; (ii) the reaction time is long, and large-scale applications cannot be realized. In order to solve the above defects, it is of great significance to fix the semiconductor photocatalyst on the carrier. Carriers such as fibers, porous nickel foam, stainless steel mesh, and porous sponge are highly valued because of their large comparative area and skeleton-like three-dimensional network structure. Among them, the fibers of semiconductor materials fabricated by the electrospinning processes have been proven to exhibit improved functional performance [26,27,28,29,30]. The technical parameters of electrospinning can be well-tuned to produce fibers with modified morphologies and mixed nanostructures. Some researchers have made studies concerning the electrospinning of oxide materials and the application of fiber materials in photocatalysis. T. Salehi et al. [26] fabricated a hybrid ZnO nanofiber/reduced graphene oxide gas sensor by electrospinning process, which showed a linear response to gas. S. Jian et al. [30] found that La-doped ZnO nanofibers were successfully fabricated via electrospinning–calcination technology, these catalysts were more efficient in rhodamine B degradation than undoped ZnO after 510 min illumination under visible light. The composite fibers were found to be stable and reusable and had potential in the management of rhodamine B dye wastewater. Meanwhile, for the photocatalysts with mixed phases, the building of multiple heterojunctions or generating some chemical defects should be more helpful to enhance the photocatalytic properties. So, how to design the multiple phases or defects involved in the composite materials and use these interfacial effects to build multiple heterojunctions should be meaningful in future experiments for researchers. This kind of photocatalyst may have great advantages and broad prospects compared to powdered catalysts.

The purpose of this study is to build multiple heterojunction structures in the photocatalysts with mixed phases of TiO_2_/BiOI composites. Especially for the synthesis process, the combined method of electrospinning and hydrothermal techniques is designed to in situ grow BiOI on the TiO_2_ fiber and make a further determination on what ratio of TiO_2_ and BiOI can have the best photocatalytic performance. The photocatalytic efficiency of TiO_2_/BiOI composite fiber was evaluated by MO degradation by visible light. Moreover, the phase tuning, Ti/Bi atomic ratio, and interfacial effects on the heterojunction structures and photocatalytic performance were studied. The related kinetics of degradation and photocatalytic mechanisms were investigated. The process in this study is simple and low in cost, which is expected to become a new method to improve the photocatalytic performance of semiconductor materials.

## 2. Materials and Methods

### 2.1. Materials

Cetyltrimethyl ammonium bromide (C_16_H_33_(CH_3_)_3_NBr) (CTAB), tetranyl titanate (C_16_H_36_O_4_Ti) (TBOT), bismuth nitrate pentachrystalline (Bi(NO_3_)_3_·5H_2_O), methyl orange (C_14_H_14_N_3_NaO_3_S) (MO) and ethylene glycol (EG) were purchased from Sinopharm Reagent Chemical Group Co., LTD (Shanghai, China). The absolute ethanol is from Shanghai Lingfeng Chemical Reagent Co., LTD (Shanghai, China). Acetic acid (CH_3_COOH) was purchased from Jiangsu Functional Chemical Co., LTD (Suzhou, China). Polyvinylpyrrolidone (PVP) was provided by Aladdin Reagent (Shanghai) Co., LTD (Shanghai, China). The purity of all reagents was above 99.7% and could be used directly in subsequent experiments. All the water used in the experiment was made by deionizing water production.

### 2.2. Electrospinning and Annealing of TiO_2_ Fibers

**Figure 1:**
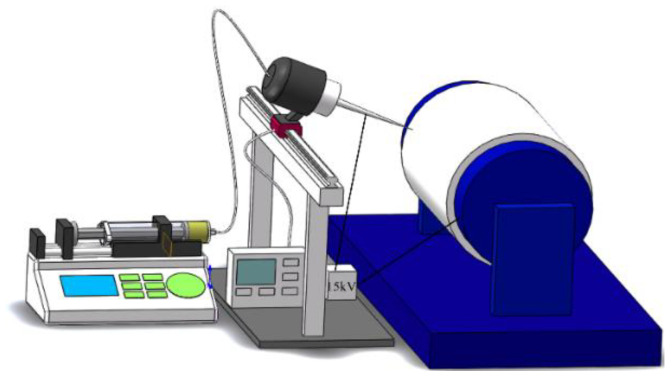
Self-made electrospinning equipment diagram.

In this study, the TiO_2_ fibers were first produced by electrospinning technology, and then the crystal structure of the fiber was regulated by heat treatment process. In the first step of spinning solution preparation, 60 g of absolute ethanol and 3 g of acetic acid were thoroughly mixed, and then 30 g of CTAB and 5 g PVP were added and stirred on magnetic stirrer for 20 min. Then, 20 g TBOT was slowly added after completely dissolving, and continued stirring until the solution is clear and transparent. When the solution appears viscous state, 10 mL was absorbed with a syringe and placed in homemade electrospinning equipment, then high voltage static electricity was applied to the liquid through 15 KV high voltage direct current. The pump speed of the homemade electrospinning machine is 1 mL/h, using an 18 # spinning needle (the outer diameter of the needle is 1.2 mm). A high voltage direct current of 15 KV was applied between the needle and the receiving plate, and the distance between the needle and the receiving plate was kept at 15 cm to ensure uniform electric field intensity. The needle was mounted on a numerical control device and reciprocated laterally at a rate of 5 cm/s, and the reciprocating movement has a period of 40 cm (in Figure 1). The fibers were spun continuously for 9 h. Under the action of electric field [31,32], charged polymer droplets can overcome the surface tension and shoot out in the form of jet stream. After the jet stream is ejected, the solvent in it will evaporate or solidify. Then, part of the solute will be deposited on the receiving device to form nonwovens such as fiber mat. The textile fiber thickness is about 200–300 nm. Finally, the prepared fibers were calcined in a Muffle furnace at 700 °C for 2 h, and then cooled naturally to room temperature with the furnace.

### 2.3. Synthesis of TiO_2_/BiOI Composite Fibers

During the experiment, 40 mL of ethylene glycol (EG) solution was added to three beakers, respectively. In order to prepare TiO_2_/BiOI composites with different proportions, 1.2/2.4/4.8 mmol Bi(NO_3_)_3_·5H_2_O was separately added to the beaker containing EG solution above, and the mixture was thoroughly mixed by magnetic stirrer. Then, 1.2/2.4/4.8 mmol of KI was separately injected, after stirring for 0.5 h, the mixed solution was poured into three reaction kettles with Teflon resin as the inner tank. At the same time, 1.2 mmol of calcined TiO_2_ fibers was added into the reaction kettle, and then the three-reaction kettle was put into the drying oven. The hydrothermal synthesis temperature was selected at 130 °C, and the holding time was 6 h. After the reactor is cooled to room temperature, discard the solution and precipitated powder in the reactor, take out the composite fiber, clean it with ethanol, and put it into the drying oven for drying at 70 °C. The obtained TiO_2_/BiOI composite fibers were sequentially labeled as T/B-1 (molar mass ratio 1:1), T/B-2 (molar mass ratio 1:2), and T/B-3 (molar mass ratio 1:4).

### 2.4. Structural Characterization

Scanning electron microscopy (FE-SEM, Hitachi S-4800II) (Hitachi, Japan) was used to study and analyze the morphology of the prepared samples. High-resolution TEM (HRTEM, Tecnai G2 F30 S-Twin) (FEI, Hillsboro, OR, USA) was used to characterize the samples at the nanoscale. The crystallization of samples was observed and characterized using D8 Advance polycrystalline X-ray diffractometer equipment (Bruker, Karlsruhe, Germany). Each sample was scanned at a speed of 0.5°/min. The valence and content of the elements were analyzed and characterized by XPS (ESCALAB 250Xi) (Thermo Scientific, Waltham, MA, USA). A Cary 5000 UV–VIS–NIR absorption spectrometer (VArian, Palo Alto, CA, USA) was used to characterize the absorbance of the samples in order to obtain the range of light absorption. The degradation efficiency of the photocatalyst was studied by electrochemical experiments on the prepared photocatalyst samples at CHI 660B workstation.

### 2.5. Photocatalytic Degradation Test

Photocatalytic performance test and analysis using the GHX-3 type photocatalytic reaction instrument purchased from Yangzhou University Science and Education City Instrument Co., Ltd (Yangzhou, China). and UV 752 type UV–visible photometer (Shanghai Yidang Analysis Co., LTD., Shanghai, China). In this study, methyl orange indicator was used as the simulated pollutant in dye wastewater, and the residual amount of methyl orange was determined by testing the absorption ability of residual methyl orange to 464 nm wavelength light. The organic wastewater simulated by methyl orange was prepared by adding 25 mg methyl orange indicator to 1000 mL deionized water at room temperature and pressure, so as to prepare 25 ppm methyl orange solution (MO).

In the photocatalytic performance test, 20 mg photocatalyst was added to the 400 mL quartz bottle of the photocatalytic reactor, and 100 mL MO solution with a concentration of 25 ppm was added to obtain the mixed suspension. Leave the suspension in the dark and turn on the magnetic force of the device for 30 min to achieve adsorption–desorption balance. Then, the 250 W xenon lamp (color temperature 6000 k) equipped with the device was turned on to irradiate the prepared suspension to simulate the visible light irradiation environment. The spacing between the light source and the sample was 8 cm. During irradiation, cooling water is supplied to the cooling water jacket of the quartz photocatalytic bottle through an external cooling water pipe. The temperature of the suspension was maintained at approximately 25 °C by cooling water, and 5 mL of the suspension was taken every 30 min. The degradation efficiency of the photocatalyst was tested by setting the reference absorbance value of 464 nm on UV–vis spectrophotometer.

## 3. Results

### 3.1. Morphologies and Crystallization

**Figure 2:**
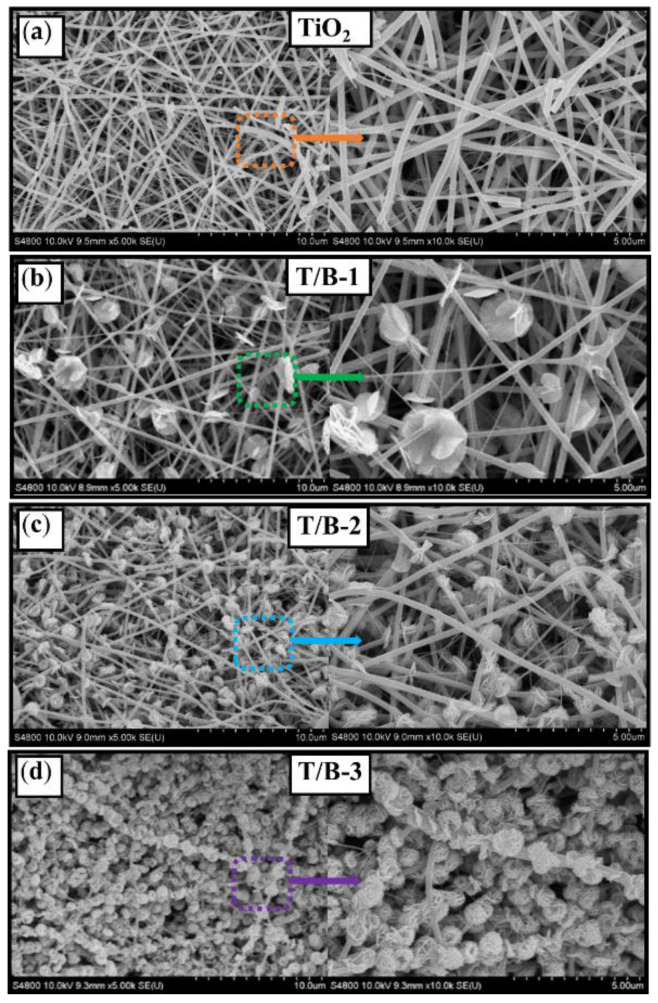
SEM images of (**a**) TiO_2_ fibers and local magnification, (**b**) T/B-1 fibers and local magnification, (**c**) T/B-2 fibers and local magnification, (**d**) T/B-3 fibers and local magnification.

The microscopic morphologies of TiO_2_, T/B-1, T/B-2, and T/B-3 fibers were characterized by scanning electron microscopy (Figure 2). For pure TiO_2_ fibers, which were arranged in disorder, showing a fluffy state, the fiber surface was smooth and clean without any impurity deposition and composite (Figure 2a). The diameter of the main body is between 200 and 400 nm, and there are a few microfibers less than 100 nm, it is caused by the instability of Taylor cone during electrospinning. The morphologies of TiO_2_/BiOI composite fibers with different Ti/Bi atomic ratios are shown in Figure 2b–d. For the sample of T/B-1, it can be observed that only a small amount of BiOI in clusters of nanosheets grown inhomogeneous on the surface of TiO_2_ fibers. With the increase in the concentration of Bi-containing precursor during solvothermal synthesis, the BiOI content on the surface of TiO_2_ fiber is constantly increased, more and more fiber surface is covered. The growth of BiOI on T/B-2 was relatively uniform, but the morphology was gradually changed to that of submicron spheres. For the sample of T/B-3, the morphology shows that the surface of TiO_2_ fiber is basically completely covered, and a large number of complete submicron spheres appeared.

Therefore, according to the above SEM observations, it can be concluded that during the solvothermal synthesis process, the BiOI morphology in situ grown on the TiO_2_ fiber surface evolved with the Ti/Bi atomic ratio in the precursor solution. It changed from nanosheets to submicron spheres structures, and the fiber surface can be totally covered by BiOI when the molar mass ratio of Ti/Bi was set at 1:4 in the precursor solution.

**Figure 3:**
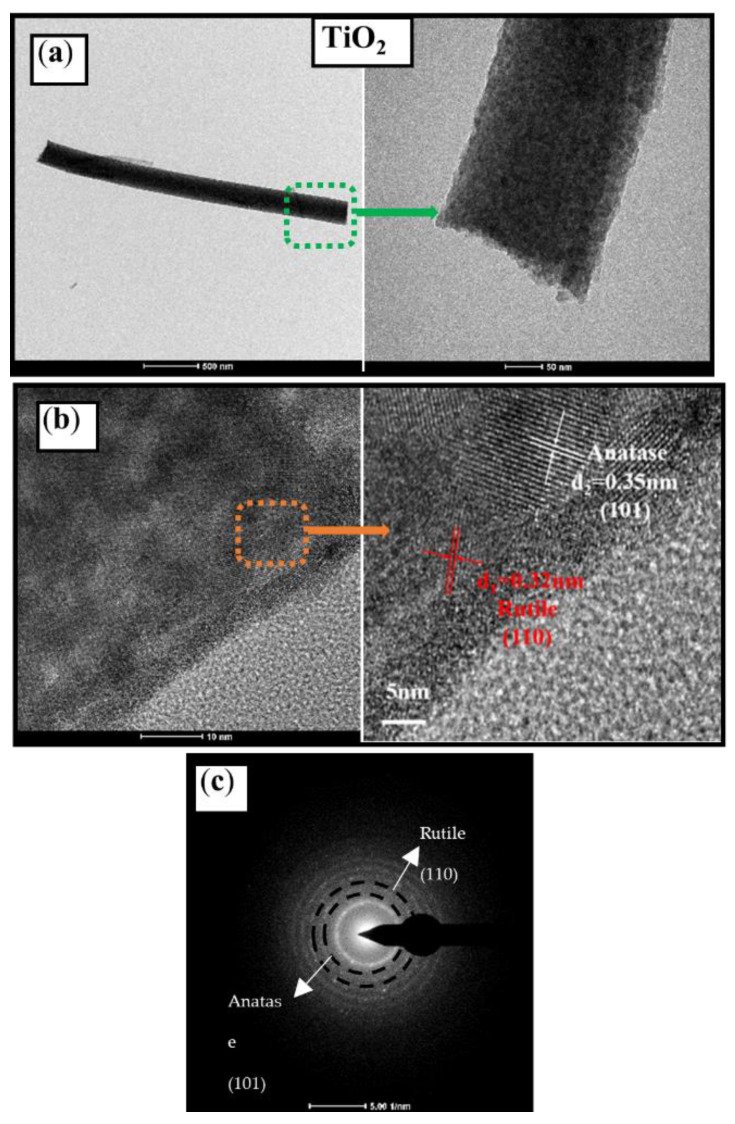
(**a**) TEM images of TiO_2_ fiber and local magnification, (**b**) HRTEM images of TiO_2_ fiber and lattice annotation of anatase and rutile, and (**c**) calibration of TiO_2_ fiber diffraction pattern.

In order to further study the microstructure and crystal structure, pure TiO_2_ and T/B-2 were analyzed in detail by TEM. In Figure 3a, it can be observed that the surface of pure TiO_2_ is flat, smooth, and compact, and there is no impurity attachment. For pure TiO_2_ samples, HRTEM (Figure 3b) and diffraction pattern (Figure 3c) was used to measure and analyze. It is found that the prepared TiO_2_ fiber exhibits a polycrystalline state after calcination at 700 °C, and the lattice spacing indicates that this polycrystalline state consists of anatase phase (0.35 nm) and rutile phase (0.32 nm). This is due to the different cooling rates at different locations of the fibers after calcination. It is further found that the two-phase structures are adjacent to each other and easy to form heterojunction structures, which may have a positive effect on photocatalytic behavior.

**Figure 4:**
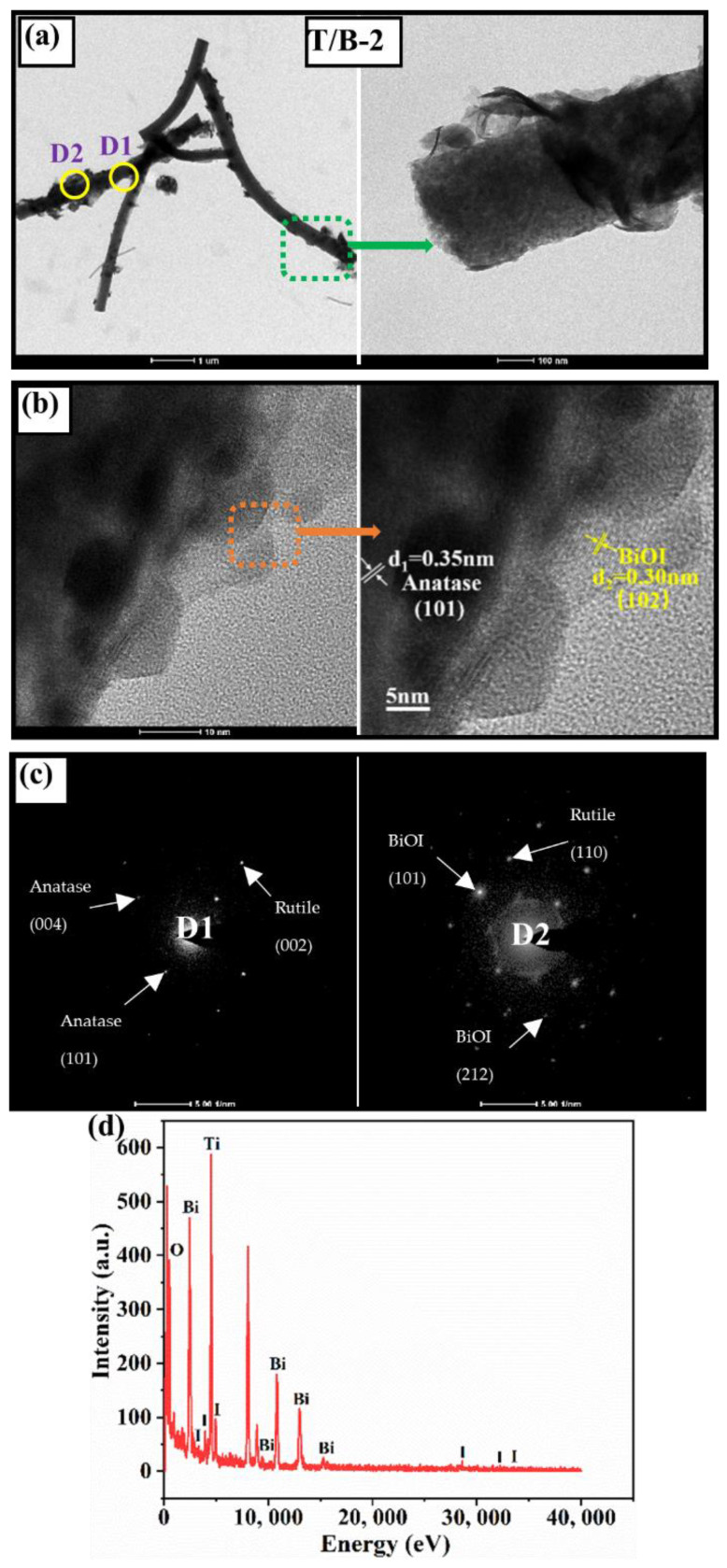
(**a**) TEM images of T/B-2 composite fiber and local magnification, (**b**) HRTEM image of T/B-2 and lattice labeling of BiOI and TiO_2_, (**d**) T/B-2 diffraction pattern calibration, (**c**) EDX map of T/B-2.

For the T/B-2 composite fiber, Figure 4a clearly shows that the flocculant distribution on the surface of TiO_2_ fiber is uniform. This is the BiOI nanosheet cluster growing on the TiO_2_ surface, and these nanosheets bind tightly to the fiber surface. HRTEM image (Figure 4b) shows that the heterojunction structure formed by the BiOI phase with lattice spacing of 0.3 nm and the anatase phase with lattice spacing of 0.35 nm [33] which should greatly improve the photocatalytic efficiency of composite fibers. Through the calibration of diffraction patterns at different positions (Figure 4c), the existence of TiO_2_ crystal phases (anatase and rutile) and BiOI crystal phases can also be found. EDX (Figure 4d) was further used to analyze the elemental composition of anatase–rutile/BiOI composite fiber. The test results indicated that the T/B-2 sample contains only Ti, O, Bi, and I, indicating that there was no contamination of other impurities in the process of sample preparation.

**Figure 5:**
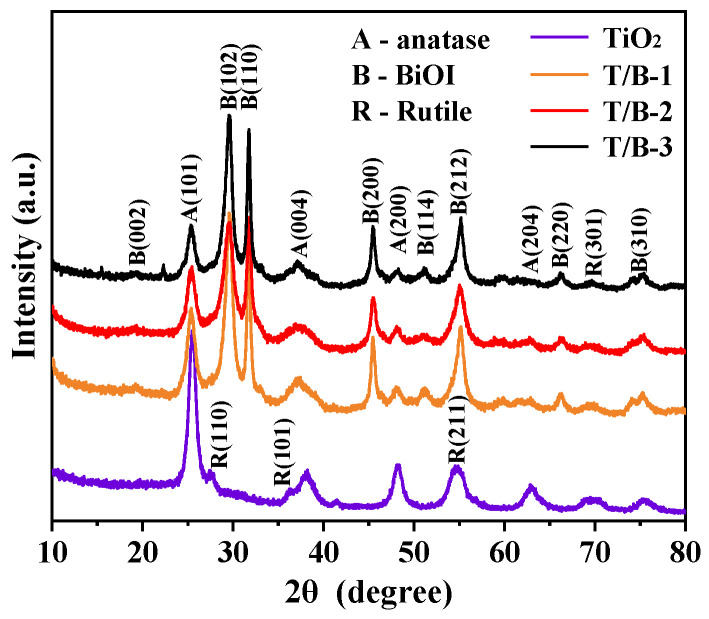
XRD patterns of TiO_2_, T/B-1, T/B-2, and T/B-3 composite fibers.

The XRD patterns of all samples are shown in Figure 5. For the crystal structure of pure TiO_2_ fiber, the anatase phase and a small amount of rutile phase are found, which is consistent with the TEM results. The anatase phase is mainly located around 25.8°, 37.8°, 48.0°, and 62.7°, corresponding to (101), (004), (200), and (204) crystals, respectively [34]. Rutile phase is mainly located at 27.4°, 36.1°, 54.3°, and 69.0°, corresponding to (110), (101), (211), and (301) crystal planes, respectively [35], and no diffraction peaks of other impurities are detected.

The XRD results of composite fibers with different proportions of BiOI and TiO_2_ show that only two characteristic peaks of BiOI and TiO_2_ phases can be identified, and no other new substances were generated. For the measured BiOI diffraction peak signals located at 19.3°, 29.6°, 31.6°, 45.3°, 55.1°, 51.3°, 66.1°, and 75.1°, corresponding to crystal planes (002), (102), (110), (200), (212), (114), (220), and (310), respectively, this matches well with the phase of BiOI (PDF#10-0445) [36]. This indicates that BiOI has been successfully synthesized on TiO_2_. With the continuous increase in BiOI concentration, the phases of TiO_2_ anatase and rutile gradually weakened, and the fibers were basically covered, which was consistent with the morphology observed by SEM.

**Figure 6:**
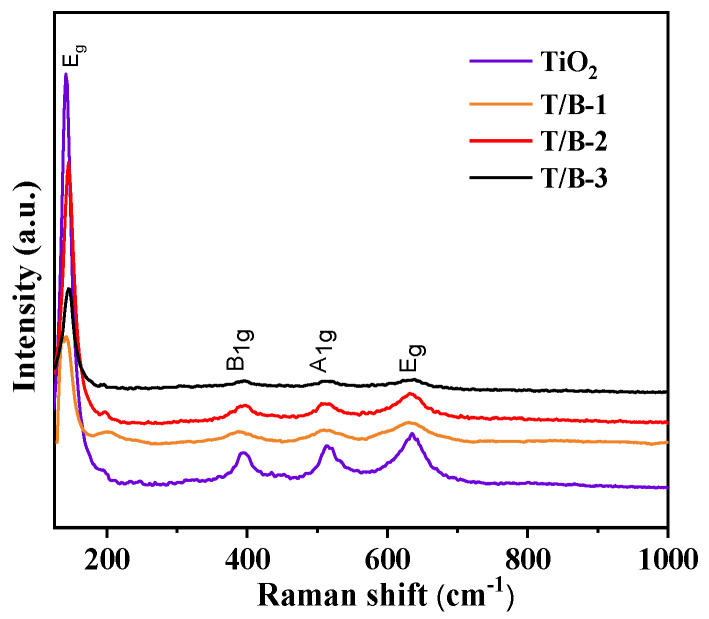
Raman spectra of TiO_2_, T/B-1, T/B-2, and T/B-3 fibers.

From the above analysis, it can be basically judged that when the concentration of BiOI precursor reaches the composite condition of T/B-2, a relatively good composite morphology is formed. In order to further confirm the generation of heterojunction structures, Raman test is needed to detect TiO_2_, T/B-1, T/B-2, and T/B-3 fibers, the results are shown in Figure 6.

The Raman active modes of anatase of TiO_2_ are located around 145 cm^−1^ (E_g_), 394 cm^−1^ (B_1g_), 518 cm^−1^ (A_1g_), and 639 cm^−1^ (E_g_), respectively [37]. Raman spectra of pure TiO_2_ samples can prove that it has the characteristics of the anatase phase. With the increase in the concentration of BiOI on TiO_2_, the E_g_ characteristic peak of TiO_2_ in composite fiber gradually weakened, which means that the shading effect of the BiOI nanosheet cluster on TiO_2_ fiber was enhanced. However, when the precursor ratio of TiO_2_ to BiOI is 1:2, the peak value of T/B-2 rises again, because BiOI can be well compounded on TiO_2_ fiber when the ratio of TiO_2_ to BiOI was 1:2. The submicron sheet clusters would not interfere with each other, and TiO_2_ should not be excessively shaded by BiOI, which can be observed by SEM images. With the increase in the concentration of BiOI, the E_g_ characteristic peak of TiO_2_ at 143 cm^−1^ has a red shift to 141.3 cm^−1^, and the E_g_ peak at 636.3 cm^−1^ has a blue shift to 638.1 cm^−1^, this indicates that the lattice structure of TiO_2_ has been distorted [38], and lattice distortion is the main factor for the generation of heterojunctions.

From the above crystallization analysis, it is concluded that the phases of anatase, rutile, and BiOI have been identified in the composite fibers, while these phases are in close contact with each other. So, the interfacial effect should be helpful to form the anatase–rutile/BiOI multiple heterojunctions in the photocatalysis of mixed-phase TiO_2_/BiOI.

### 3.2. Chemical Structure

**Figure 7:**
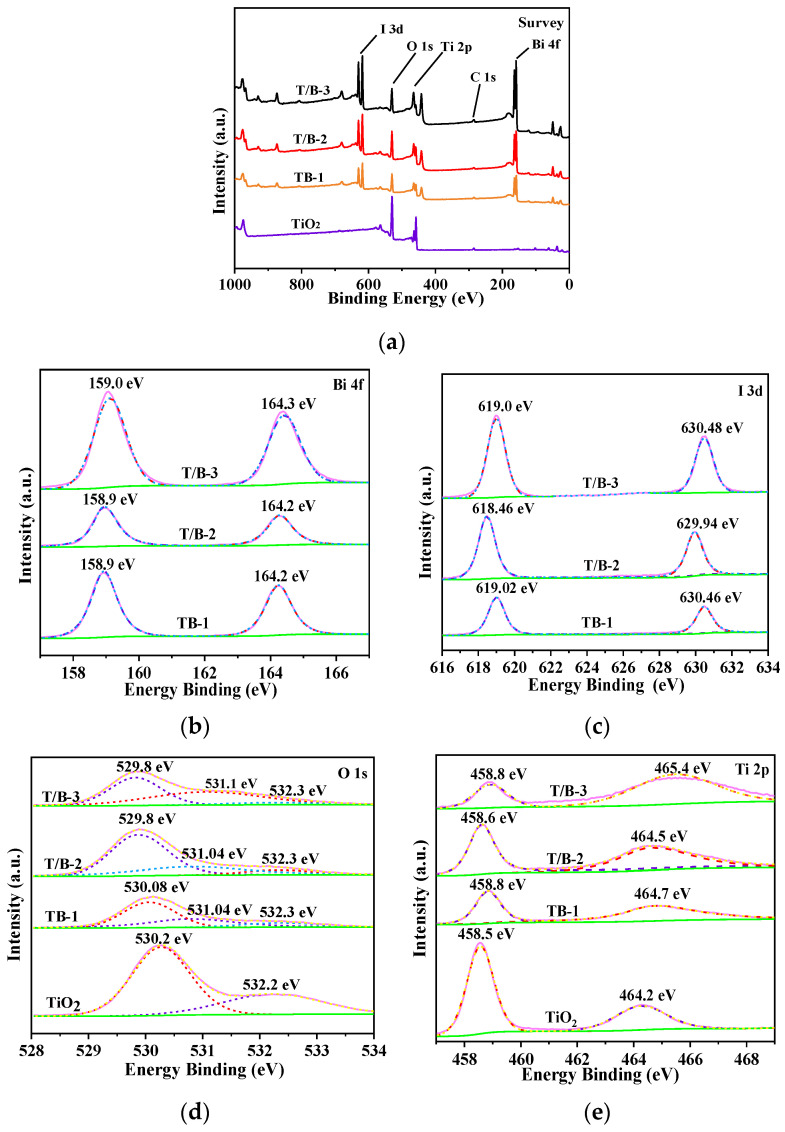
XPS spectra of TiO_2_, T/B-1, T/B-2 and T/B-3 composite fibers, (**a**) survey spectra, (**b**) Bi 4f spectra, (**c**) I 3d spectra, (**d**) O 1s spectra, and (**e**) Ti 2p spectra.

The chemical element composition and chemical bonding state of TiO_2_, T/B-1, T/B-2, and T/B-3 fibers were analyzed and tested by XPS. Ti, Bi, O, and I can all be verified in the XPS survey curves in Figure 7a. The element contents of the four samples are shown in Table 1. In the full spectrum of XPS (Figure 7a), it can be identified that the pure TiO_2_ fiber surface is only a Ti peak and O peak, there is no other impurity peak.

With the continuous addition of BiOI, Bi and I peaks appeared, and the peaks of Bi and I gradually increased. The characteristics of Bi^+^ in BiOI can be observed in the detailed XPS spectrograms of Bi 4F (Figure 7b), namely the Bi 4F_7/2_ peak near 158.9 eV and the Bi 4F_5/2_ peak near 164.3 eV [39]. In Figure 7c, the peaks of I 3d_5/2_ and I 3d_3/2_ binding energies are located around 618.7 eV and 630.2 eV, respectively, which also obviously reflects that I^−^ is from BiOI [40]. Figure 7d shows that the peaks corresponding to 530.2 eV and 532.2 eV before recombination are characteristic peaks of Ti-O and H_2_O, respectively. The three peaks formed after recombination are Ti-O (around 529.8 eV), Bi-O (around 531.1 eV), and O-H (532.3 eV) [41]. In Figure 7e, The peak of Ti 2P_1/2_ is located near 464.4 eV and Ti 2p_3/4_ is located near 458.6 eV, which proves the existence of Ti^4+^ [42]. After recombination, the peak of Ti 2P_1/2_ shifts to the direction of higher binding energy by at most 1.3 eV, which again proves the formation of a heterojunction structure. It can be found from Table 1 that with the continuous increase in BiOI concentration, the content of the Ti element decreased significantly, while the content of the Bi element gradually increased, and the value of Ti/Bi decreased significantly, which was consistent with the situation observed by SEM and XRD.

**Figure 8:**
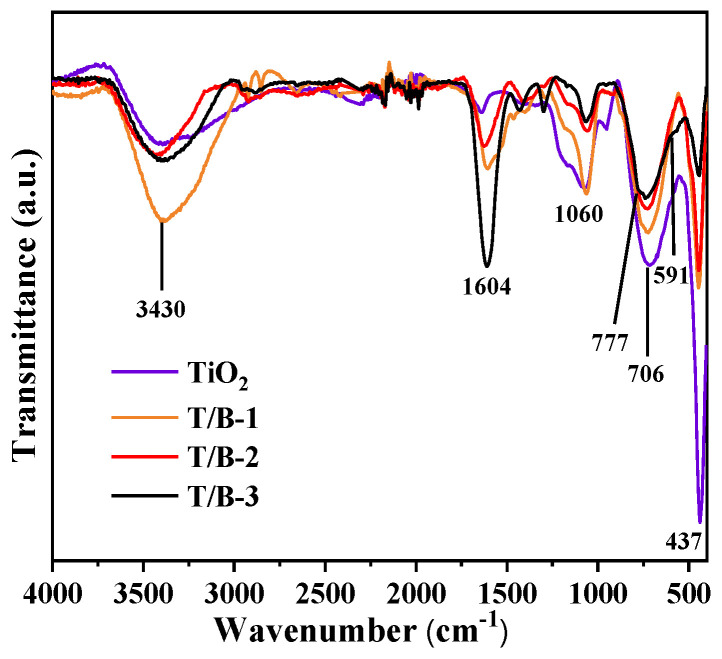
FTIR detection spectra of TiO_2_, T/B-1, T/B-2, and T/B-3 composite fibers.

Subsequently, FTIR spectroscopy was used to characterize the chemical structure of TiO_2_, T/B-1, T/B-2, and T/B-3 fibers, as shown in Figure 8. In the TiO_2_ spectrum, the peak width in the wide frequency band of 500–800 cm^−1^ corresponds to the peak produced by the Ti-O-Ti bending vibration of TiO_2_. A total of 1604 cm^−1^ and 3430 cm^−1^ are caused by the bending vibration of adsorbed H_2_O and -OH. The peak at 3000 cm^−1^ is caused by the presence of O-H [43,44]. With the addition of BiOI and the continuous increase in compound concentration, the characteristic peak of the anatase phase at 437 cm^−1^ was hidden with the increase in the number of BiOI nanosheet clusters, and the peak intensity decreased significantly. The polar hydroxyl group (-OH) attached to the heterojunction between TiO_2_ and BiOI was vibrating, so the characteristic peak at 1604 cm^−1^ was significantly enhanced [45]. The characteristic peak at 1051 cm^−1^ is the wide peak centered by the vibration of the Ti–OH bond. As BiOI continues to attach to TiO_2_ fibers, the shading effect is enhanced, the fracture peak width of the Ti–OH bond is narrowed [46], and there is an obvious blue shift, which is influenced by the heterojunction between TiO_2_ and BiOI. This is consistent with the TEM observation (Figure 4b).

### 3.3. Calculation of UV–Vis Spectra and Bandgaps

**Figure 9:**
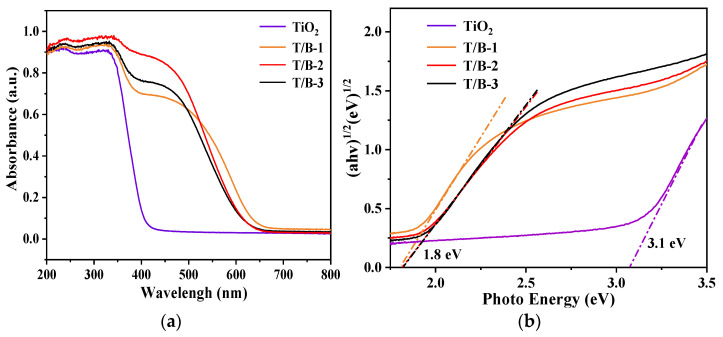
(**a**) UV–vis spectra of composite fibers prepared from hydrothermal solutions with different components, (**b**) bandgap diagram of composite fibers prepared by hydrothermal solution with different composition.

An important factor affecting the absorption performance of semiconductors is the width of the band gap. The narrower the band gap, the wider the absorption range. UV–vis absorption was used to test TiO_2_, T/B-1, T/B-2, and T/B-3 fibers, and the absorption curves were shown in Figure 9a. However, in the visible light wavelength range, it can be found that TiO_2_ fiber almost does not absorb. When BiOI was compounded on the surface of TiO_2_, heterojunction was formed, and the absorption of visible light was significantly improved. It indicates that TiO_2_-BiOI interfacial effect leads to a low band gap value. This is because of the unique multilayer structure of BiOI, which still has some unpaired electrons inside. Free electron binding on the surface of TiO_2_ may lead to the formation of, for example, a Ti–O–Bi bond, which makes the valence band move up. Finally, the composite fiber appears to have a smaller band gap [47].

The following reference formula is often used to calculate the optical bandgap of photocatalysts:(ahν)^1/n^ = C(hν − E_g_) [48], BiOI is an indirect bandgap semiconductor with index *n* = 2 [49]. The calculated band gap value is shown in Figure 9b. The E_g_ value of TiO_2_ is 3.1 eV, and the values for T/B-1, T/B-2, and T/B-3 are all around 1.8 eV. Obviously, compared with pure TiO_2_ fiber, the band gap values of the composite fibers are significantly reduced, which is helpful to generate more photogenerated carriers and has a great impact on the improvement of photocatalytic efficiency.

### 3.4. Photocatalytic Performance Test

**Figure 10:**
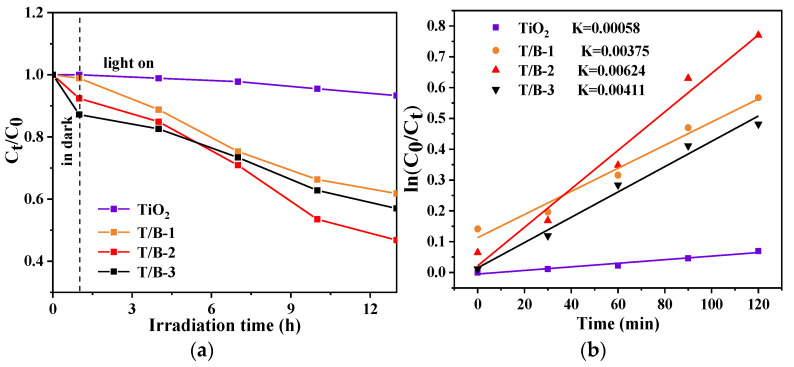
(**a**) Photocatalytic degradation efficiency of methyl orange by different fibers, (**b**) first-order kinetics of photodegradation of methyl orange.

The degradation capacity of TiO_2_, T/B-1, T/B-2, and T/B-3 fibers was simulated by using methyl orange solution in a photocatalytic reactor. Figure 10a shows the degradation capacity of different fibers. It can be observed that pure TiO_2_ fiber has the most unsatisfactory degradation effect on methyl orange solution, the T/B-2 composite fiber has the best degradation rate (reached 54%), and the degradation rate of T/B-3 was slightly lower than that of T/B-2, which may be caused by the inability of TiO_2_ fiber to absorb more photogenerated electrons from BiOI. Although T/B-1 composite fiber has a narrow band gap, it is not effective in photocatalytic test, this may be attributed to the fragility of BiOI in the form of micron sheets, during the photocatalytic test, BiOI was impacted by water flow, and the heterojunction [50] could not work normally.

The photocatalytic performance of the prepared composite in this work is compared with the experimental results of other authors (in Table 2), it can be seen that the prepared anatase–rutile/BiOI composite fibers have better degradation efficiency relative to the recent literature review on TiO_2_/BiOI photocatalysts. In order to further explore the optical absorption properties of TiO_2_, T/B-1, T/B-2, and T/B-3 fibers, empirical formulas were used to fit the measured data [51,52]. Figure 10b shows that the degradation rate constant K values of TiO_2_, T/B-1, T/B-2, and T/B-3 fibers are 0.00058, 0.00375, 0.00624, and 0.00411 min^−1^, respectively. The photodegradation rate of T/B-2 is higher than that of other fibers, about 12 times that of pure TiO_2_. This is because the obtained band gap is narrow, and the electron holes are not easy to aggregate.

**Figure 11:**
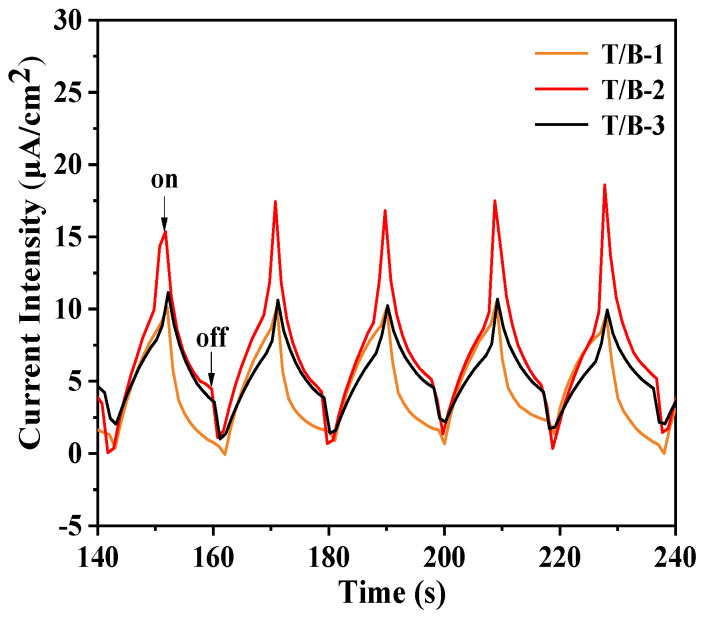
Transient photocurrent response profiles of T/B-1, T/B-2, and T/B-3 composite fiber heterojunctions.

In the experiment, the photocurrent test method was also used to characterize the separation efficiency of electron holes, as shown in Figure 11. The detected T/B-1, T/B-2, and T/B-3 composite fibers all had photocurrent responses, and the value of T/B-2 was found to be increased obviously. The results show that when the composite precursor ratio is 1:2, the charge transfer efficiency of the sample is higher, which well proves the accuracy of the experimental results of photocatalytic degradation.

Briefly, the degradation reaction of methyl orange is through the excitation of photogenerated electrons and band gaps, and the holes generated after excitation react with pollutants. Moreover, the direction of electron transfer between TiO_2_/BiOI photocatalyst is determined by the conduction band (CB) and valence band (VB) of BiOI and TiO_2_. However, CB and VB of BiOI and TiO_2_ need to be calculated by the following formula [56]:

E_VB_ = X − E_C_ + 0.5E_g_ (1)

E_CB_ = E_VB_ − E_g_ (2)

where X represents the absolute electronegativity of semiconductor photocatalytic materials, Ec is the free electron energy under hydrogen label. E_g_ stands for bandgap value, which is obtained from the UV–Vis test. The band gap values for BiOI, anatase TiO_2_, and rutile TiO_2_ were 1.74 eV, 3.1 eV, and 3.0 eV, respectively. Table 3 shows the CB and VB values of BiOI, anatase TiO_2_, and rutile TiO_2_, respectively.

It can be seen that the TiO_2_ fiber surface attached to BiOI can reduce the band gap value. Therefore, it is easy to generate more photogenerated electrons and holes under simulated sunlight, which is conducive to improving the photocatalytic ability of pure TiO_2_ under sunlight.

**Figure 12:**
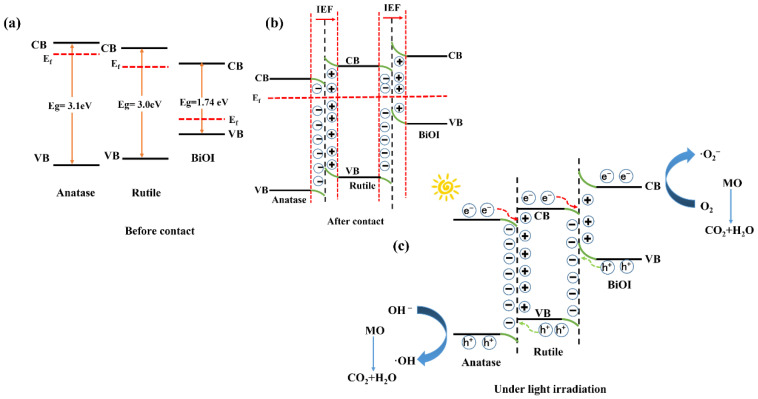
Charge transfer process in anatase–rutile TiO_2_/BiOI S-type heterojunction: (**a**) before contact, (**b**) after exposure, (**c**) transfer of photogenerated carriers under light irradiation.

Based on the above analysis, anatase–rutile/BiOI conforms to the charge transfer process of S-type heterojunction structure, as shown in Figure 12 [57]. Before contact, the CB and Fermi levels of rutile TiO_2_ and anatase TiO_2_ are above BiOI. The work function (Φ) of rutile TiO_2_, anatase TiO_2_, and BiOI are calculated using empirical function ∆ V = Φ − φ [58,59]. ∆ V represents the contact potential difference and φ is the work function of the XPS test instrument itself. After calculation, the work functions of rutile TiO_2_, anatase TiO_2_, and BiOI are 6.31 eV, 6.51 eV, and 8.57 eV, respectively. The work function is inversely proportional to the Fermi level. Fermi energy levels of rutile TiO_2_ and anatase TiO_2_ are close. BiOI with the maximum work function has the lowest Fermi energy level, as shown in Figure 12a. When anatase TiO_2_ and rutile TiO_2_, rutile TiO_2_ and BiOI are in full contact [60], Fermi level can reach equilibrium. At this time, the work functions of rutile TiO_2_, anatase TiO_2_, and BiOI change to 6.22 eV, e^−^ on BiOI will spontaneously diffuse to the surface of TiO_2_ and form the accumulation layer and consumption layer of e^−^ on the surface (Figure 12b) [61]. At the same time, an IEF is generated at the contact point. In the process of contact (Figure 12c), The Fermi levels of anatase TiO_2_, rutile TiO_2_, and BiOI move up and down, respectively, which is the fundamental reason for the band bending up and down. IEF, coulomb force, and band bending will recombine photogenerated electrons with holes, and IEF also plays a role in accelerating the combination. This improves the efficiency of anatase–rutile/BiOI photocatalysis.

**Figure 13:**
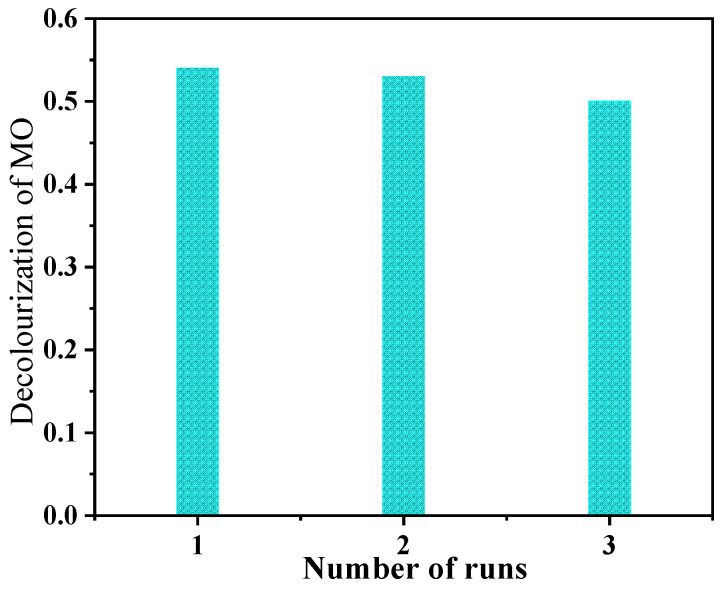
Cyclic testing of photocatalytic performance of T/B-2.

Subsequently, we tested the photocatalytic results of the T/B-2 composite fibers after recycling (in Figure 13). First, the composite fibers were filtered from MO solution and rinsed repeatedly with absolute ethanol and deionized water. Then, put the rinsed fibers into the drying box to dry them again. After that, the photocatalytic test was carried out for 150 min and repeated twice. The results show that the degradation efficiencies of the first two times are not changed so much, the performance is slightly reduced in the third cycle, which may be due to the shedding of BiOI caused by water flushes.

## 4. Conclusions

In summary, BiOI was successfully synthesized on the surface of TiO_2_ fiber by the combination of electrospinning and hydrothermal synthesis processes. The study concerning the Ti/Bi atomic ratio of TiO_2_/BiOI composite fiber on the nanostructures and related photocatalytic performance was conducted. Variable morphologies of the composite fibers have been obtained according to [Ti/Bi], which is changed from nanosheets to submicron spheres structures, and the fiber surface can be totally covered by BiOI when the molar mass ratio of Ti/Bi was set at 1:4 in the precursor solution. The crystallization including anatase, rutile, and BiOI has been evidenced, and the chemical state of the Ti element was shifted for interfacial effects which should be helpful to form the anatase–rutile/BiOI multiple heterojunctions in the photocatalysis of mixed-phase TiO_2_/BiOI. The photocatalytic activity of the synthesized TiO_2_/BiOI composite fiber was improved 10~300% compared with the results from the literature, it also can reach about 12 times the degradation efficiency of the original TiO_2_ fiber. This should be caused by the formation of multiple heterojunction structures of anatase–rutile/BiOI composite fibers. The multiple heterojunction structures have enhanced the absorption of visible light and reduced the band gap value, which leads to the increase in photocurrent. Moreover, the S-type heterojunction mechanism is supposed to increase the degradation rate under visible light irradiation, which induces the separation of photogenerated electrons and holes. The process in this study is simple and low in cost, which is expected to become a new method to improve the photocatalytic performance of semiconductor materials.

## Figures and Tables

**Table 1:** Summary of elements contents of TiO_2_, T/B-1, T/B-2, and T/B-3 composite fibers.

Samples	Element Concentration				Ti/Bi
	Ti (%)	O (%)	Bi (%)	I (%)	
TiO_2_	21.90	64.41	0	0	/
T/B-1	12.94	57.07	7.51	8.54	1.71
T/B-2	13.59	61.99	8.63	5.64	1.57
T/B-3	9.89	54.12	9.65	11.54	1.02

**Table 2:** The recent literature review on TiO_2_/BiOI and their photocatalytic efficiency under visible light illumination.

Photocatalysts	Preparation Methods	Simulated Pollutant	The Degradation Rate Constant K Value	Ref
BiOI/TiO_2_/ZIF-8	Hydrothermal synthesis	Norfloxacin (NOR)	0.0058 min^−1^	[53]
TiO_2-x_/BiOI/AgBr	Hydrothermal synthesis	Methylene blue (MB)	0.0033 min^−1^	[54]
BiOI/TiO_2_	Electrostatic self-grouping induced by surface charge	MO	0.0026 min^−1^	[55]
* Anatase–rutile/BiOI	Hydrothermal synthesis	MO	0.0062 min^−1^	/

* Represents the photocatalysts synthesized in this study.

**Table 3:** VB and VC potentials corresponding to BiOI and TiO_2_.

Materials	E_g_ (eV)	E_VB_ (eV)	E_CB_ (eV)
BiOI	1.74	2.36	0.62
Rutile TiO_2_	3.00	2.84	−0.16
Anatase TiO_2_	3.10	2.89	−0.21

## Data Availability

The data presented in this study are available on request from the corresponding author.
